# Investigating health service availability and readiness for antenatal testing and treatment for HIV and syphilis in Papua New Guinea

**DOI:** 10.1186/s12884-022-05097-w

**Published:** 2022-10-19

**Authors:** Olga PM Saweri, Neha Batura, Justin Pulford, M. Mahmud Khan, Xiaohui Hou, William S Pomat, Andrew J Vallely, Virginia Wiseman

**Affiliations:** 1 The Kirby Institute, University of New South Wales, Sydney, Australia; 2 The Papua New Guinea Institute of Medical Research, Goroka, Papua New Guinea; 3 Institute for Global Health, University College London, London, UK; 4 Liverpool School of Tropical Medicine, Liverpool, UK; 5 University of Georgia, Athens, USA; 6 The World Bank Group, Washington, DC USA; 7 Department of Global Health and Development, London School of Hygiene and Tropical Medicine, London, UK

**Keywords:** Health facility readiness, Antenatal care, HIV, Syphilis, Health service assessment, Pacific

## Abstract

**Background:**

Papua New Guinea (PNG) has one of the highest burdens of HIV and syphilis in pregnancy in the Asia-Pacific region. Timely and effective diagnosis can alleviate the burden of HIV and syphilis and improve maternal and newborn health. Supply-side factors related to implementation and scale up remain problematic, yet few studies have considered their impact on antenatal testing and treatment for HIV and syphilis. This study explores health service availability and readiness for antenatal HIV and/or syphilis testing and treatment in PNG.

**Methods:**

Using data from two sources, we demonstrate health service availability and readiness. Service availability is measured at a province level as the average of three indicators: infrastructure, workforce, and antenatal clinic utilization. The readiness score comprises 28 equally weighted indicators across four domains; and is estimated for 73 health facilities. Bivariate and multivariate robust linear regressions explore associations between health facility readiness and the proportion of antenatal clinic attendees tested and treated for HIV and/or syphilis.

**Results:**

Most provinces had fewer than one health facility per 10 000 population. On average, health worker density was 11 health workers per 10 000 population per province, and approximately 22% of pregnant women attended four or more antenatal clinics. Most health facilities had a composite readiness score between 51% and 75%, with urban health facilities faring better than rural ones. The multivariate regression analysis, when controlling for managing authority, catchment population, the number of clinicians employed, health facility type and residence (urban/rural) indicated a weak positive relationship between health facility readiness and the proportion of antenatal clinic attendees tested and treated for HIV and/or syphilis.

**Conclusion:**

This study adds to the limited evidence base for the Asia-Pacific region. There is a need to improve antenatal testing and treatment coverage for HIV and syphilis and reduce healthcare inequalities faced by rural and urban communities. Shortages of skilled health workers, tests, and medicines impede the provision of quality antenatal care. Improving service availability and health facility readiness are key to ensuring the effective provision of antenatal care interventions.

**Supplementary Information:**

The online version contains supplementary material available at 10.1186/s12884-022-05097-w.

## Background

Sexually transmitted infections (STIs) are associated with adverse pregnancy and birth outcomes including miscarriage, preterm birth, low birthweight, stillbirth, and neonatal death [1–11]. Globally, 1.4 million pregnant women are diagnosed with HIV, and 988 000 with syphilis annually; most infections occur in low- and middle- income countries (LMICs) [12]. Papua New Guinea (PNG) has one of the highest burdens of HIV and syphilis in the Asia-Pacific region [13]. Studies indicate that approximately half of all pregnant women in PNG are infected with not just one, but multiple STIs, including HIV and syphilis [14].

Timely and effective diagnosis and treatment can help alleviate the high burden of STIs among pregnant women [14, 15]. Implementation and scale-up of these interventions remain a major challenge for many LMICs. For HIV and syphilis, the introduction of serological single-use point-of-care test kits has made timely and accurate detection feasible, and is a widely accepted part of routine antenatal care (ANC) [16]. Many LMICs, including PNG, have integrated HIV and syphilis testing and treatment into routine ANC. However, there is a growing body of evidence highlighting significant structural barriers to delivering these services at scale [17], such as a lack of diagnostics, medicines, an inadequate number and mix of skilled health workers, deteriorating health facility infrastructure, and financing or budgetary constraints [18–20]. These barriers can hinder the provision of quality ANC in LMIC, including the consistent testing and appropriate treatment for HIV and syphilis in pregnancy [21–24].

HIV and syphilis testing have been part of routine ANC in PNG for over 10 years [16, 25]. A national health indicator surveillance report estimated that approximately 51.3% of antenatal clinic attendees were tested for HIV in 2019 [26] and 44.2% for syphilis in 2018 [27]. Further, HIV and syphilis prevalence in pregnancy in 2019 was 1.6% and 4% respectively [28], resulting in the persistent occurrence of adverse pregnancy and birth outcomes [26, 29]. Over the last decade, there has been little to no change in antenatal testing coverage for HIV and syphilis in PNG, which has indirectly affected treatment rates [26, 29]. As a result, the government of PNG has set a national target for antenatal testing and treatment for HIV and syphilis coverage, which is 90% of antenatal attendees by 2030 [26].

Timely and appropriate testing and treatment of HIV and syphilis at scale requires the availability of appropriate human and capital resources to provide quality health services, meet national coverage targets, and achieve better health outcomes. It has become increasingly common to use service availability and readiness tools to identify gaps in service delivery [30]. This results in an evaluation of health system performance or specifically an assessment of health system capacity. This study aimed to evaluate health service availability and health facility readiness for antenatal testing and treatment for HIV and syphilis in PNG. Additionally, this study explored the associations between health facility readiness and antenatal testing and treatment of HIV and syphilis in PNG. To our knowledge, this is the first study of its kind, using nationally representative survey data, in the Pacific region.

## Methods

### Study setting

PNG has a population of more than 8 million people, 85% of whom live in rural areas [31]. Administratively, PNG is divided into 22 provinces across four geographical regions, namely Momase, Highlands, Southern and the New Guinea Islands. Public healthcare is decentralised and funded through budgetary allocations from the national and provincial governments, donor agencies, non-governmental organizations, church-groups and private service providers [32].

Health facilities in PNG are categorised hierarchically according to size and clinical capability. Health services are decentralised by province, where there are six levels, and each level is defined by a set of minimum standards, such as staff ceilings and service provision [33]. Levels 1-4 focus on primary health care whilst levels 5-7 provide secondary healthcare in addition to primary health care. Table 1 summarises the key characteristics of health facilities at each level.

**Table 1:** Health facility characteristics by type of health facility^a^

**Health facility type**	**Number of health facilities**	**Average number of health workers**^**b**^	**Average number of beds**^**c**^	**Average catchment population **[32]
**Primary healthcare (levels 1-4)**				
** Community health posts**	3088^d^	3 (2)	1.9	500-2000
** Sub-health centers and urban clinics**	458^d^	6.4 (4)	5.4	2000-5000 (rural population) >10 000 (urban population)
** Health centers**	261^d^	11 (7)	16.5	5000 -10 000
** District/rural hospitals**	14	39.5 (14-35)	61.3	>70 000
**Secondary healthcare (levels 5-7)**				
** Provincial/ / national referral hospitals**	21^e^	176 (63-355)	78.8	1000- total population

^a^Source: [34]

^b^Indicates the observed average number of health workers, and in parentheses are the number of mandated health workers according to National Health Service Standards. Source: [32]

^c^Source: [27]

^d^Not all health facilities are open (operational) or staffed

^e^Includes twenty provincial hospitals, and one national referral hospital

### Data sources

Data from two sources were used for the analysis. The first source was the national health indicator surveillance (NHIS) system [27], retrieved from the National Department of Health (NDoH) in September 2020. The NHIS is a monitoring and surveillance instrument showcasing year-on-year progress towards health sector medium- and long-term targets [25, 35]. Privately owned/managed health facilities and pharmacy-based clinics are not part of the NHIS dataset, and therefore not included in this study [30]. We used data collected from 813 health facilities in 2015 to ensure compatibility with the second data source (described below). From the NIHS dataset, we obtained the number of health facilities per province, the size of the provincial health workforce, antenatal clinic attendance or utilization per province, the number of antenatal clinic attendees tested for HIV and syphilis per health facility, and the seropositive antenatal clinic attendees treated for HIV per health facility. Data for the number of seropositive antenatal clinic attendees treated for syphilis per health facility was not available, and therefore not included in this analysis.

The second data source was a national health facility efficiency survey completed in 2015 [36]. The survey was jointly funded by the NDoH, the Australian Department of Foreign Affairs and Trade (DFAT) and the World Bank and carried out by the Arnold School of Public Health of the University of South Carolina, the Nossal Institute of Public Health of the University of Melbourne, and the PNG Institute of Medical Research. The survey was undertaken in 73 public and church-run health facilities to investigate efficiencies in healthcare delivery. The sample included all publicly run hospitals (level 5-7 health facilities) and followed a two-step sampling procedure for level 3-4 health facilities. Firstly, six to eight districts were randomly selected in 13 provinces across all four regions. Secondly, a purposive selection of the largest level 3-4 health facilities were selected. A more detailed explanation of the study design can be obtained from the survey’s full report [36]. All health facilities included this sample provide ANC services, including antenatal testing and treatment for HIV and syphilis. Data on health facility amenities, infection prevention protocols, and the availability of diagnostic equipment, medicines and medical supplies was extracted from this dataset to calculate health facility readiness scores. To investigate the association between the proportion of antenatal clinic attendees tested for HIV and syphilis, and the seropositives treated, we matched variables retrieved from the NHIS data with the national health facility efficiency survey.

### Defining key variables

Service availability demonstrates the physical presence of health services across a country’s health system [22, 30]. This study explores service availability using the World Health Organization’s (WHO) SARA toolkit. The SARA toolkit measures service availability by three indicators related to infrastructure, health workers and health service utilisation [30, 37, 38], which we extracted from the NHIS [27]:

- Density of health facilities: the number of operational health facilities per 10 000 population by province. This includes all public and church-run health facilities (*N*=813) reported in the NHIS.
- Density of core health workers: the number of core health workers per 10 000 population by province. This includes all clinician cadres, namely medical officers (doctors), health extension officers (HEOs)^1^, nurses, midwives, and community health workers (CHWs)^2^ [39].
- Antenatal clinic utilization: the percentage of pregnant women making four or more visits to an antenatal clinic, by province. This is based on the number of births and stillbirths reported as a proxy for the number of pregnant women (measure used by the NDoH [35]).

Health facility readiness is measured by the factors related to a health facility’s capacity to provide healthcare [22, 30]. This study specifically measures the readiness to provide routine ANC services. Adopting the approach used by Leslie et al. (2017) [40], we created a health facility readiness score for each facility that comprised four domains: amenities; protocols for infection prevention; diagnostic equipment; and medicines and medical supplies Each domain includes multiple indicators (see Table 2). The choice of indicators for each domain was guided by the SARA toolkit [30] and adapted to the PNG context [16]. A full description of how the scores were compiled is presented below under ‘statistical analysis’.

**Table 2:** Domains and indictors of the supply service readiness for HIV and syphilis testing and treatment

**Domain / Indicators**	**Explanation of indicator**
**Basic amenities**	
** Electricity**	Electricity is available
** Back-up generator**	Back-up power source (generator) available
** Fuel for generator**	Health facility has fuel to run the generator
** Running water**	Running water is available and piped into the health facility
** Toilet**	At least one functional toilet (can be pit without slab, pit with slab or flushable) available for patients
** Communication equipment**	A functional telephone (landline), shortwave radio or mobile phone is available for communication purposes
** Emergency transport**	A vehicle, not necessarily an ambulance, is available and has the primary function of transporting patients
**Protocols for infection prevention**	
** Sharps final disposal**	Protocol in place for final disposal of sharps (e.g., taken of offsite, burnt and/or buried)
** Final bio-waste disposal**	Protocol in place for final disposal of bio-waste (e.g., taken offsite, burnt and/or buried)
** Waste segregation**	Segregates sharps, general- and bio-waste into different bins
**Diagnostic equipment**	
** EDD calculator**	Expected date of delivery (EDD) calculator available and able to estimate EDD and gestational age
** Scale**	Available to measure the weight of antenatal clinic attendees
** Tape measure**	Available to measure fundal height (size of uterus) and able to estimate gestational age
** Foetal stethoscope**	Pinard horn is available to check the baby’s heart rate
** Blood pressure machine**	Sphygmomanometer is available to measure blood pressure
** Refrigerator/Vaccine carrier**	Suitable vaccine carrier available to store/administer vaccines safely
** Urinalysis dipsticks and collection cups**	In-stock and available to test urine for protein and glucose to help diagnose pre-eclampsia and gestational diabetes
** Point-of-care HIV test-kits**	Serological HIV point-of-care test-kits to test for HIV are available and in-stock (including 30 days prior to survey)
** Point-of-care syphilis test-kits**	Serological syphilis point-of-care test-kits to test for syphilis are available and in-stock (including 30 days prior to survey)
**Medicines and medical supplies**	
** Gloves**	Examination gloves are available and in-stock (including 30 days prior to survey)
** Needles and syringes**	Needles and syringes are available and in-stock (including 30 days prior to survey)
** Cotton wool**	Cotton wool is available and in-stock (including 30 days prior to survey)
** Water for injection**	Water for injection, used to reconstitute substances, is available and in-stock (including 30 days prior to survey)
** Iron and folate supplement**	Iron and Folate oral supplement, used to treat anaemia, is available and in-stock (including 30 days prior to survey)
** Sulfadoxine/pyrimethamine (SP)**	SP, used for intermittent prevention of malaria in pregnancy, is available and in-stock (including 30 days prior to survey)
** Tetanus toxoid**	Tetanus toxoid, used to prevent neonatal tetanus, is available and in-stock (including 30 days prior to survey)
** Benzathine-penicillin**	Benzathine-Penicillin, used to treat syphilis, is available and in-stock (including 30 days prior to survey)
** Antiretroviral therapy (ART)**	ART, first line therapy used to treat HIV, is available and in-stock (including 30 days prior to survey). ART comprises of tenofovir or zidovudine plus lamivudine and efavirenz or nevirapine

### Statistical analysis

The service availability score is the average of three indicators: infrastructure; workforce; and antenatal clinic utilization, and is expressed as a percentage [30]. Each indicator is calculated at the province level and calculated using the following equations.

**Infrastructure:** density of health facilities per 10 000 population (shown by province); compared to the global WHO target of 2 health facilities per 10 000 population [30].

1 $$\begin{array}{c}\mathrm{Provincial}\\ \mathrm{Infrastructure}\end{array}=\left({~}^{\left(\frac{\text{Number of Open Health Facilities}}{\left(\mathrm{Population}\div10 000\right)}\right)}\! \left/ \!{~}_{\text{WHO Target}}\right. \right)\times 100\mathrm{\%}$$

**Workforce:** density of core health workers per 10 000 population (shown by province); compared to the global WHO target of 45 health workers per 10 000 population [30, 39].

2 $$\begin{array}{c}\mathrm{Provincial}\\ \mathrm{Workforce}\end{array}=\left({~}^{\left(\frac{\text{Number of Core Health Workers}}{\left(\mathrm{Population}\div10 000\right)}\right)}\! \left/ \! {~}_{\text{WHO Target}}\right. \right) \times 100\mathrm{\%}$$

**Antenatal clinic utilization:** proportion of pregnant women making at least 4 antenatal clinic visits per province [41]. Antenatal clinic utilization is compared to the global target of all pregnant women having at least 4 antenatal clinic visits per province [42]. The WHO now recommends at least 8 antenatal clinic visits per pregnancy [41]; however, this study utilises the target of at least 4 antenatal clinic visits per pregnancy as per national policy [25].

3 $$\begin{array}{c}\mathrm{Provincial}\\ \mathrm{ANC\;Utilization}\end{array}=\left(\frac{\mathrm{No}.\;\mathrm{Pregnant \;Women\;Accessing\;ANC\;at\;Least }\;4\mathrm{\;Times}}{\mathrm{No}.\;\mathrm{of\;Pregnant\;Women}}\right)\times 100\mathrm{\%}$$

Service availability is the average of Eqs. 1, 2, and 3:

4 $$\mathrm{Service Availability }(\mathrm{\%})= \frac{(\mathrm{Infrastructure}+\mathrm{Workforce}+\mathrm{ANC utilization}) }{3}$$

Health facility readiness is a composite score comprising 28 indicators across four domains (Table 2). Each indicator is dichotomous with a value of 0 demonstrating the indicator is not available and a value of 1 indicating it is available, multiplied by the domain weight and presented as a percentage. We initially used principal component analysis (PCA) to determine data-derived weights and construct readiness scores. To demonstrate the sample’s suitability for a PCA, we ran a Kaiser-Meyer-Olkin (KMO) test for sampling adequacy. The KMO test indicates whether there is sufficient correlation within a sample to conduct a PCA or factor analysis [43, 44]. A KMO score greater than 0.5 suggests that the variables within a sample are highly correlated and justifies the use of PCA [44–46]. The KMO score for this sample was 0.3, thus the use of the PCA was not justified. Given this, equal weights were attached to each domain and to each indicator across the four domains. The use of equal weights is common practice in health readiness analyses [18, 30, 40, 47, 48]. A score of 100 indicates that a health facility has all the necessary components to effectively test and treat antenatal clinic attendees for HIV and syphilis at point-of-care. The equation is shown below:

5 $$\begin{array}{c}\mathrm{Readiness }\\ \mathrm{score}\end{array}=\left(0.25\left(\mathrm{amenities}\right)+ 0.25\left(\begin{array}{c}\mathrm{infection}\\ \mathrm{prevention}\end{array}\right)+0.25\left(\begin{array}{c}\mathrm{antenatal}\\ \mathrm{equipment}\end{array}\right)+0.25\left(\begin{array}{c}\mathrm{antenatal}\\ \mathrm{medicines}\end{array}\right)\right)\times 100\mathrm{\%}$$

In the linear regression, data from 73 facilities surveyed were used to assess the association between testing and treating HIV and syphilis in an antenatal clinic with health facility readiness. The main dependent variables were:

- Proportion of antenatal clinic attendees tested for HIV;
- Proportion of seropositive antenatal clinic attendees treated for HIV; and
- Proportion of antenatal clinic attendees tested for syphilis.

Independent variables included in the regression analysis were selected based on the published literature [18, 40, 47] and available data at the health facility level:

- Health facility type: classification of a health facility as a health centre, district hospital or a provincial hospital;
- Health facility residence: located in either a rural or an urban setting;
- Managing authority: public- or church -run health facilities;
- Catchment population: identified by a health facility as the population it serves; and
- Clinicians employed: the number of medical officers, nursing officers, HEOs and CHWs working at a health facility.

For the bivariate analysis, the proportion of antenatal clinic attendees tested for HIV was regressed on health facility readiness using a linear regression, with robust standard errors (Eq. 6). Thereafter, conditional on antenatal clinic attendees being tested for HIV, we regressed the proportion of seropositive antenatal clinic attendees treated for HIV (Eq. 7). The equations were:

6 $$\begin{array}{c}\mathrm{proportion of }\\ \mathrm{antenatal clinic}\\ \mathrm{ attendees tested for HIV}\end{array}= {\upbeta }_{0}+{\upbeta }_{1 }\left(\begin{array}{c}\mathrm{health facility}\\ \mathrm{readiness}\\ \mathrm{score}\end{array}\right)+\mathrm{U}$$

7 $$\begin{array}{c}\mathrm{proportion of HIV}\\ \mathrm{seropositive antenatal clinic}\\ \mathrm{ attendees treated}\end{array}={\upbeta }_{0}+{\upbeta }_{1 }\left(\begin{array}{c}\mathrm{health facility}\\ \mathrm{readiness}\\ \mathrm{score}\end{array}\right)+\mathrm{U}$$

Due to a lack of treatment data for syphilis, it was only possible to explore the association between the proportion of antenatal clinic attendees tested for syphilis and health facility readiness (Eq. 8). The regression equation for syphilis was:

8 $$\begin{array}{c}\mathrm{proportion of }\\ \mathrm{antenatal clinic attendees}\\ \mathrm{ tested for syphilis}\end{array}={\upbeta }_{0}+{\upbeta }_{1 }\left(\begin{array}{c}\mathrm{health facility}\\ \mathrm{readiness}\\ \mathrm{score}\end{array}\right)+\mathrm{U}$$

In the multivariate analysis, all dependent variables were continuous, while the independent variables were both continuous and categorical. The health facility readiness score, the number of clinicians employed (on a logarithmic scale) were continuous, while health facility residence (urban or rural), type of health facility, management of the health facility (public- or church-run), and catchment population were all categorical. When fitting the model, the ‘residence’ variable was excluded due to collinearity. We used *P*<0.05 to determine statistical significance. The linear regression equations, with robust standard errors for testing HIV (Eq. 9) and treating seropositives for HIV (Eq. 10) were as follows:

9 $$\begin{array}{c}\mathrm{proportion of }\\ \mathrm{antenatal clinic attendees}\\ \mathrm{ tested forHIV}\end{array} ={\upbeta }_{0}+{\upbeta }_{1 }\left(\begin{array}{c}\mathrm{health facility}\\ \mathrm{readiness}\\ \mathrm{score}\end{array}\right)+{\upbeta }_{2 }\left(\begin{array}{c}\mathrm{management}\\ \mathrm{authority}\end{array}\right)+{\upbeta }_{3 }\left(\begin{array}{c}\mathrm{health}\\ \mathrm{facility}\\ \mathrm{type}\end{array}\right)$$

10 $$\begin{array}{c}\mathrm{proportion of HIV}\\ \mathrm{seropositive antenatal clinic}\\ \mathrm{ attendees treated}\end{array}={\upbeta }_{0}+{\upbeta }_{1 }\left(\begin{array}{c}\mathrm{health facility}\\ \mathrm{readiness}\\ \mathrm{score}\end{array}\right)+{\upbeta }_{2 }\left(\begin{array}{c}\mathrm{management}\\ \mathrm{authority}\end{array}\right)+{\upbeta }_{3 }\left(\begin{array}{c}\mathrm{health}\\ \mathrm{facility}\\ \mathrm{type}\end{array}\right)$$

The regression equation for syphilis (Eq. 11) was as follows:

11 $$\begin{array}{c}\mathrm{proportion of }\\ \mathrm{antenatal clinic attendees}\\ \mathrm{ tested for syphilis}\end{array}={\upbeta }_{0}+{\upbeta }_{1}\left(\begin{array}{c}\mathrm{health facility}\\ \mathrm{readiness}\\ \mathrm{score}\end{array}\right)+{\upbeta }_{2}\left(\begin{array}{c}\mathrm{management}\\ \mathrm{authority}\end{array}\right)+{\upbeta }_{3}\left(\begin{array}{c}\mathrm{health}\\ \mathrm{facility}\\ \mathrm{type}\end{array}\right)+{\upbeta }_{4}\left(\mathrm{log}\begin{array}{c}\mathrm{clinicians}\\ \mathrm{employed}\end{array}\right)+{\upbeta }_{5}\left(\begin{array}{c}\mathrm{catchment}\\ \mathrm{population}\end{array}\right)+\mathrm{U}$$

All statistical analyses were carried out using Microsoft Excel version 365 (Microsoft Corp) and Stata version 13 (Stata Corp).

## Results

All 73 health facilities in the sample provided ANC services and were included in this analysis. Table 3 provides a summary of health facilities and service statistics. Most health facilities included in this sample were health centres (64.4%) and located in rural areas. All hospitals were in urban townships, or provincial towns. Most health facilities in this sample were public health facilities (64.4%). All hospitals were publicly run, while just under half of the district hospitals and health centres were church run (48%). Antenatal HIV testing coverage was lower in urban health facilities (5.6%) compared to rural health facilities (23.5%), while HIV treatment coverage was lower in rural health facilities (1.1%) relative to urban health facilities (4.2%). The average proportion of antenatal clinic attendees tested for syphilis was higher in urban health facilities (11.1%) relative to rural health facilities (4.3%).

**Table 3:** Summary of health facilities (*N*=73) and service statistics

**Variable**	**Health facilities n (%)**
**Region**	
** Highlands**	21 (28.8)
** Momase**	17 (23.3)
** New Guinea Islands**	17 (23.3)
** Southern**	18 (24.7)
** Health facility type**	
** Hospital**	19 (26)
** District/ Rural Hospital**	7 (9.6)
** Health Centre**	47 (64.4)
**Location**	
** Urban**	19 (26)
** Rural**	54 (74)
**Managing authority**	
** Public**	47 (64.4)
** Church**	26 (35.6)
** Population, and service statistics**	Average (range)
**Pregnant women in catchment population (n)**	
** Urban**	1560 (116- 14 408)
** Rural**	627 (36- 2273)
**Pregnant women making at least one antenatal clinic visit (n)**	
** Urban**	1043 (64- 3206)
** Rural**	295 (29- 857)
**Antenatal clinic attendants tested for HIV at sampled health facilities (%)**	
** Urban**	5.6 (0- 38.9)
** Rural**	23.5 (0- 100)
**HIV seropositive antenatal clinic attendants treated at sampled health facilities (%)**	
** Urban**	4.2 (0- 50)
** Rural**	1.1 (0- 40)
**Antenatal clinic attendants tested for syphilis at sampled health facilities (%)**	
** Urban**	11.1 (0- 81.7)
** Rural**	4.3 (0- 28.4)
**Syphilis seropositive antenatal clinic attendants treated at sampled health facilities (%)**	
** Urban**	No data
** Rural**	No data

### Service availability

**Fig. 1:**
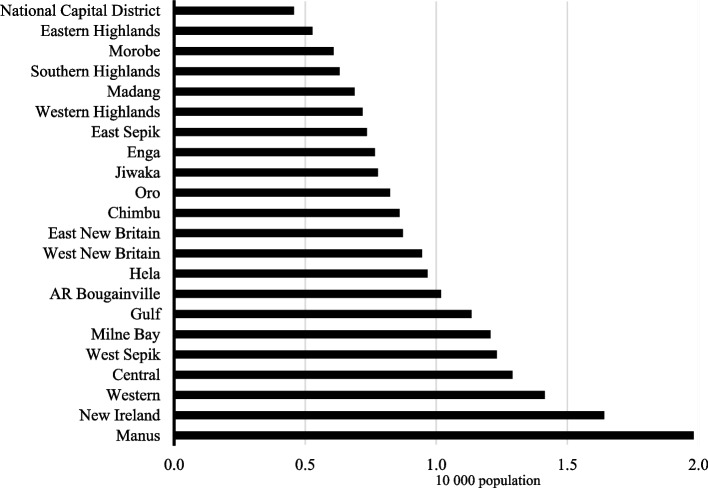
Infrastructure - Density of health facilities per province in PNG

Figure 1 demonstrated that only one province, Manus, achieved the WHO health facility density target. Most provinces (14 out of 22 or 63.6%) had fewer than one health facility per 10 000 population. The two provinces with the lowest density of open health facilities were the National Capital District and Eastern Highlands Province, both had 0.5 open health facilities per 10 000 population.

**Fig. 2:**
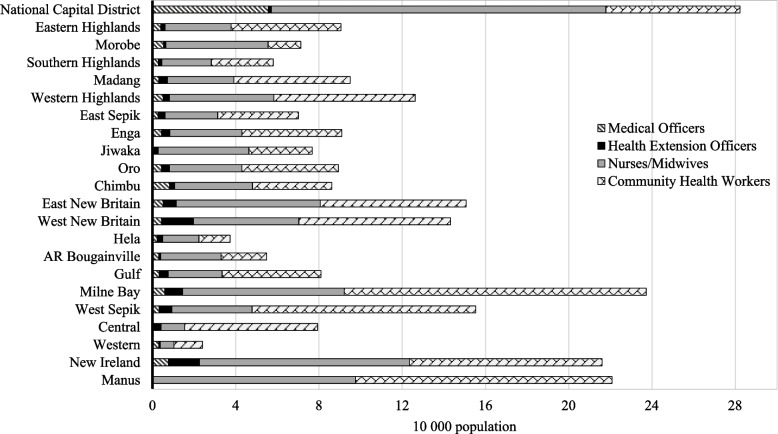
Workforce - Density of core health workers per province in PNG

Figure 2 showed that no provinces met the health worker density target. In every province, the bulk of the healthcare workforce was made up of nurses, midwives, and CHWs. On average, there was less than one medical officer per 10 000 population per province; the largest share of medical officers work in the nation’s capital which had on average 5.6 medical officers per 10 000 population. Central province and Jiwaka did not report any medical officers employed.

**Fig. 3:**
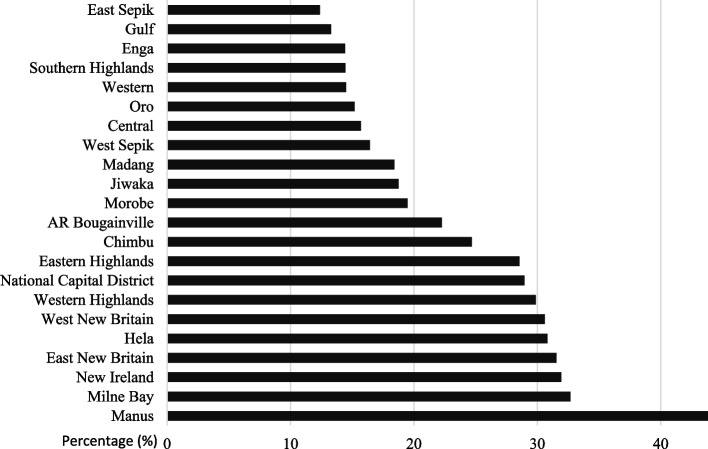
Antenatal clinic utilisation - Percentage of pregnant women having four or more antenatal clinic visits per province in PNG

Figure 3 demonstrated that on average, 22% of pregnant women attended four or more antenatal clinics in 2015. No provinces met the target of all pregnant women attending four or more antenatal clinics. Further, 11 of 22 (50%) provinces had fewer than 20% of pregnant women attending at least four antenatal clinics throughout pregnancy. ANC utilization was highest in Manus, where about 46% of pregnant women attended at least four antenatal clinics throughout pregnancy.

**Fig. 4:**
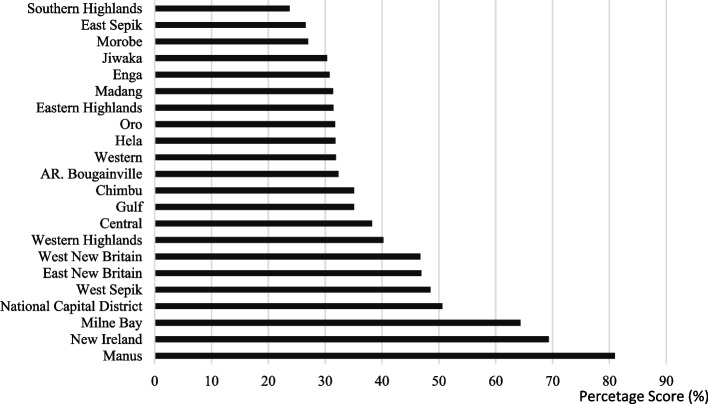
Service availability score per province in PNG

Only 4 out of 22 provinces had a service availability score above 50% (Fig. 4). Service availability ranged from 24% in the Southern Highlands to 81% in Manus, the national average was 40%. Further, 12 of 22 (55%) provinces, had a service availability score between 30 and 40%.

### Health facility readiness

**Fig. 5:**
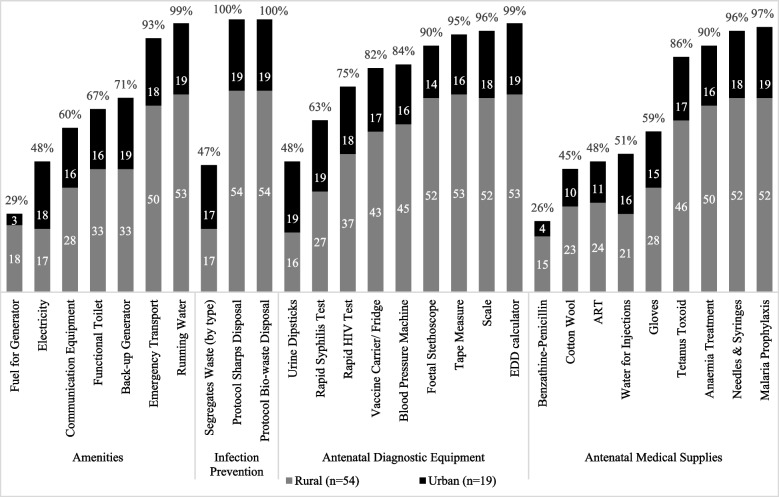
Health facility readiness: indicators present at all sampled health facilities

Average health facility readiness was 66%, and most health facilities scored between 51 and 75%. Figure 5 illustrates the availability of indicators that make up the readiness score segregated by rural and urban health facilities. With respect to amenities, nearly all health facilities reported having running water (piped directly into the health facility by local authorities or a water tank reliant on rain collection) and emergency transport. Not all health facilities had functioning toilets (61% of rural health facilities and 84% of urban). Only 30% of rural health facilities were connected to the main power grid, compared to 90% of urban health facilities. However, 61% rural facilities had a generator and 33% fuel for a generator, which facilitates electricity supply in these areas. All facilities had capacity for infection prevention, and disposed of waste appropriately, either by burning and/or burying, using an incinerator, or disposing off-site. Diagnostic equipment across all facilities, including test kits for protein and glucose and point-of-care test kits for HIV and syphilis, were less likely to be available in rural health facilities compared to urban ones. Lastly, with respect to the availability of drugs, the treatment for HIV and syphilis, Antiretroviral therapy (ART) and benzathine penicillin respectively, were not adequately supplied across all health facilities sampled (Fig. 5).

**Fig. 6:**
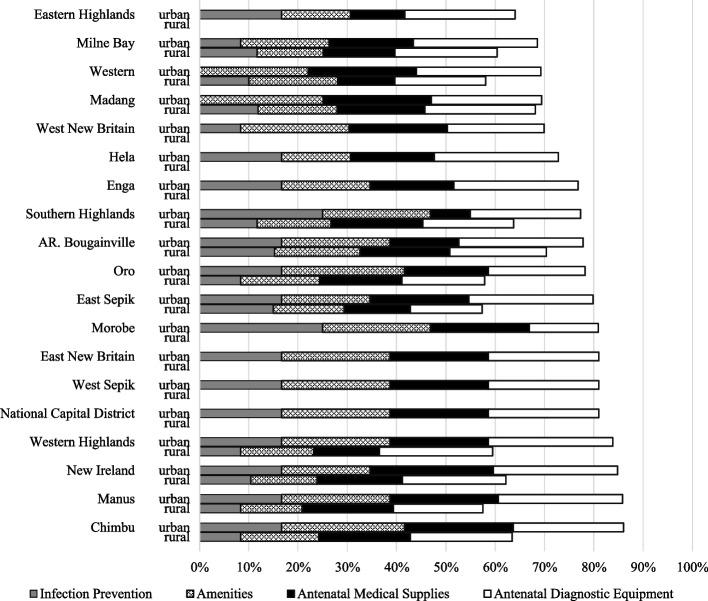
Health facility readiness (expressed as a percentage score) by province

Figure 6 illustrates health facility readiness per province. Average health facility readiness for urban health facilities was 77% (95% CI: 0.721-0.802), while for rural health facilities it was 62% (95% CI: 0.591-0.680). All health facilities scored above 50%, although none achieved a score of 100%. In general, urban health facilities had a greater capacity to provide antenatal point-of-care testing and treatment for HIV and syphilis compared to rural ones.

### Regression analysis

Table 4 presents the results of the bivariate robust linear regressions. There were positive relationships between health facility readiness and the proportion of antenatal clinic attendees tested and treated for HIV, as well as the proportion of antenatal clinic attendees tested for syphilis. However, none of the relationships were found to be significant (*p*-value = 0.05).

**Table 4:** Bivariate association between health facility readiness and the proportion of antenatal clinic attendees tested and treated for HIV and Syphilis

**Proportion of antenatal clinic attendees tested for HIV**^**a**^			
	**Coefficient [95% CI]**	**t**	***P*> |t|**
Supply-side readiness score	12.78 [0- 59.67]	0.54	0.589
**Proportion of HIV seropositive antenatal clinic attendees treated**^**b**^			
	**Coefficient [95% CI]**	**t**	***P*> |t|**
Supply-side readiness score	10.25 [0 – 21.95]	1.75	0.085
**Proportion of antenatal clinic attendees tested for Syphilis**^**c**^			
	**Coefficient [95% CI]**	**t**	***P*> |t|**
Supply-side readiness score	22.7140 [0 – 46.14]	1.93	0.057

^a^Tested for HIV: Number of observations: 73; R-squared: 0.0042; Prob>F: 0.5885; RMSE: 26.196

^b^Treated for HIV: Number of observations: 73; R-squared: 0.0276; Prob>F: 0.085; RMSE: 8.1432

^c^Tested for syphilis: Number of observations: 73; R-squared: 0.0563; Prob>F: 0.0573; RMSE: 12.433

CI: Confidence Interval; *P*> |t|: *P*-value of t-statistic; Prob>F: probability of F statistic; RMSE: Root Mean Square Error; t: t-statistic

Table 5 presents the results of all three multivariate regressions. Health facility readiness was not associated with antenatal testing and treatment for HIV or antenatal testing for syphilis (*p*-value= 0.05). However, the size of the catchment population was shown to be associated with antenatal HIV and syphilis testing, while health facility was only associated with HIV testing

**Table 5:** Multivariate association between health facility readiness and the proportion of antenatal clinic attendees tested and treated for HIV and syphilis

**Variable**	**Tested for HIV**^**a**^			**HIV seropositives treated**^**b**^			**Tested for Syphilis**^**c**^		
	**Coefficient [95% CI]**	**t**	***P*> |t|**	**Coefficient [95% CI]**	**t**	***P*> |t|**	**Coefficient [95% CI]**	**t**	***P*> |t|**
**Readiness score**	29.59 [0 – 87.06]	1.03	0.307	-3.12 [0 – 16.62]	-0.32	0.753	-0.26 [0 – 16.94]	-0.03	0.976
**Health facility type**									
** Hospital**	Reference Category								
** District/rural hospital**	35.05 [0 – 75.73]	1.72	0.090	-0.67 [0 – 9.00]	-0.14	0.890	2.17 [0 – 15.67]	0.32	0.749
** Health centre**	24.38 [1.75 – 47.02]	2.15	0.035	1.07 [0 – 10.43]	0.23	0.820	-5.42 [0 – 10.03]	-0.70	0.486
**Public/ Church run**									
** Church**	Reference Category								
** Public**	8.83 [0 – 23.30]	1.22	0.227	3.07 [0 – 8.10]	1.22	0.228	0.62 [-0– 5.09]	0.28	0.783
**Size of catchment**									
** >10 000**	Reference Category								
** 10 000 – 19 999**	8.85 [0 – 24.41]	0.55	0.260	5.67 [0 – 11.75]	1.86	0.067	7.14 [0 – 14.28]	2.00	0.050
** 20 000 – 29 999**	22.17 [8.51 – 35.84]	2.71	0.002	-0.62 [0 – 5.21]	-0.21	0.833	8.07 [0 – 17.34]	1.74	0.087
** 30 000 – 49 999**	2.53 [0 – 19.24]	1.00	0.763	-0.52 [0 – 6.56]	-0.15	0.883	-4.71 [0 – 2.46]	-1.31	0.194
** < 50 000**	22.23 [0 – 46.50]	2.04	0.072	1.51 [0 – 8.74]	0.42	0.678	-6.86 [0 – 1.80]	-1.58	0.118
** Clinicians employed**	1.14 [0 – 9.95]	0.30	0.768	2.13 [0 – 5.42]	1.29	0.202	2.11 [0 – 5.63]	1.20	0.236

^a^Number of observations: 72; R-squared: 0.3065; Prob>F: 0.0119; RMSE: 23.341

^b^Number of observations: 72; R-squared: 0.1354; Prob>F: 0.3914; RMSE: 8.217

^c^Number of observations: 72; R-squared: 0.2547; Prob>F: 0.0441; RMSE: 11.805

CI: Confidence Interval; *P*> |t|: *P*-value of t-statistic; Prob>F: probability of F statistic; RMSE: Root Mean Square Error; t: t-statistic

## Discussion

This study aimed to explore the service availability and health facility readiness for delivery of antenatal point-of-care HIV and syphilis testing and treatment in PNG. We identified critical gaps in health service availability and health facility readiness. Proximity to a health facility has been shown in the past to be a key determinant of accessing ANC in PNG and other LMICs [49, 50]. Only one province in our study met the global WHO target of two health facilities per 10 000 population. This signals that access to ANC remains a major challenge for PNG and a contributing factor to low antenatal utilization reported in this study and others [49–51].

Our findings demonstrated significant shortages of health workers across PNG. Health services rely heavily on nurses, midwives, and CHWs, who make up the majority of the health workforce. There is a need to increase the number of health workers, particularly medical officers, trained in PNG. Approximately 50 medical officers become fully registered practitioners annually [32], with the majority practicing in urban locations. Most, if not all, LMICs face skilled health worker shortages, and also have difficulty educating, training, deploying, and retaining health workers, especially medical officers, in rural areas [39]. This has also been a long-standing challenge for the PNG health system [36, 52]. Many strategies to address health worker deployment and retention have been implemented and evaluated across LMICs including exposing trainees to rural health care during residency [53], financial incentive programs [54], and introducing new levels of health worker cadres, such as HEOs [55]. In PNG, HEOs are a vital component of rural health care, catering for the sparse number of medical officers working in rural areas. HEO training has evolved from a certificate nearly 20 years ago into a bachelor’s degree in health science (rural health) in 2008. However, despite this training program, our results show that health worker deployment and retention in rural areas remains problematic. The government of PNG needs to establish a sustainable program targeted toward increasing the number of health workers across all cadres in rural areas to improve service provision [39, 56].

Globally, average ANC utilization is 83% [57], the results of this study demonstrate that ANC utilization in PNG is concerningly low and far below the global average. Globally, the barriers to accessing antenatal care are well-documented [58, 59], and include financial, social and cultural constraints [49]; health worker attitudes [60]; or as discussed above, geographical barriers [61]. More recently, greater attention has been paid to patient perceptions of healthcare quality [19, 40, 62–66]. For example, studies have found that women will forego seeking care at their nearest health facility and elect to attend a larger health facility due to concerns regarding quality of care [67]. ANC utilization can be improved through interventions designed to enhance expectant mothers’ knowledge and understanding of the importance of ANC [49], patient experience , interactions between ANC attendees and healthcare workers [68, 69], and the quality of ANC [50], and costs of seeking care [70].

Health facility readiness was generally low across all health facilities, although urban facilities tended to be better equipped to deliver routine antenatal testing and treatment for HIV and syphilis compared to rural health facilities. Of particular concern was the limited supply of diagnostic tests in rural health facilities compared to urban ones, while limited stock of treatment for HIV and syphilis was a problem across all health facilities sampled. The results indicate ailing health facility infrastructure and poorly equipped health facilities, which are commonly reported in LMICs [20, 71]. These results illustrate the difficulties associated with health facility operations, the quality of service provision, and general health facility maintenance under constrained budgets [72]. Strategies, including year-on-year health facility maintenance, and strengthening procurement and distribution of diagnostics, medicines, and medical supplies, are required to meet the demand for health services in PNG.

The regression results showed that antenatal HIV and/or syphilis testing and treatment were weakly associated with health facility readiness. Factors that had a statistically significant association with testing and treatment were the size of the catchment population and the type of health facility. Similar studies recognize the association between supply-side factors, such as health facility readiness, and testing and treating for HIV and syphilis [18, 73, 74]. Thus, future research that includes additional variables, such as trained staff and guidelines, and a larger sample would improve our understanding of the relationship between supply side readiness and antenatal testing and treatment for HIV and syphilis in PNG.

A key strength of our study was the use of two nationally representative datasets that enabled the identification of major variations in readiness scores in 73 health facilities across 19 of PNG’s 22 provinces. Further, through data linkage, we were able to explore for the first time, associations between clinical outcomes (testing and treatment for HIV and testing for syphilis) and health facility readiness. Our study also had some limitations. First, health facility readiness provides only a partial view of the quality of antenatal testing and treatment for HIV and syphilis in PNG. However, it is a strong starting point for identifying the inputs necessary to deliver quality antenatal testing and treatment for HIV and syphilis. Second, the association between health facility readiness and the proportion of antenatal clinic attendees treated for syphilis was not included in this analysis. This was because none of the 73 health facilities included in the regression analysis reported any syphilis treatment data in the NHIS. Despite this, we believe that the results presented using the HIV treatment data are indicative of the relationship between health facility readiness and treatment for syphilis. This is due to the similarities in both structure and funding for the national HIV and syphilis programs in PNG [26, 75]. Lastly, the data was retrieved from routinely reported NHIS data is limited to availability. The data are reported at the health facility level, and data quality and integrity may differ between health facilities. However, as the study team was involved in data collection and quality checks, we are confident of its accuracy.

## Conclusion

The results from this study add to the extremely limited literature on supply side factors affecting the delivery of quality ANC in the Pacific. They highlight a need to improve antenatal testing and treatment coverage for HIV and syphilis, particularly inequalities between rural and urban communities. Shortages in the availability of tests and treatments for HIV and syphilis as well as in skilled health workers, are of particular concern and hinder universal access to quality ANC. Basic amenities such as electricity also need to be improved. There is potential for the PNG health system to increase rates of testing and treatment for HIV and syphilis in pregnancy by improving facility readiness to provide these services. We recommend that future studies investigate the quality of antenatal care delivered at rural and urban facilities in PNG, which would complement the results presented in this study.

## Supplementary Information

**Additional file 1.** STROBE Statement—Checklist of items that should be included in reports of cross-sectional studies Title of the study: Investigating health service availability and readiness for antenatal testing and treatment for HIV and syphilis in Papua New Guinea.

## Data Availability

The datasets generated and/or analysed during the current study are not publicly available as they contain several identifying variables and openly sharing the data would compromise the privacy of participating health facilities. However, the data are available from the corresponding author on reasonable request.

## Abbreviations

ANC: Antenatal care
ART: Antiretroviral therapy
CHW: Community health worker
DFAT: Australian Department of Foreign Affairs and Trade
EDD: Expected delivery date.
HEO: Health extension officer
HIV: Human immune deficiency virus
KMO: Kaiser-Meyer-Olkin test
LMIC: Low- and middle- income country
MBBS: Bachelor of Medicine and Bachelor of Surgery
NDoH: National Department of Health
NHIS: National Health Indicator Surveillance
PCA: Principal component analysis
PCR: Polymerase chain reaction
PNG: Papua New Guinea
SARA: Service Availability and Readiness Analysis
SP: Sulfadoxine/pyrimethamine
STI: Sexually transmitted infection
WHO: World Health Organization

## Footnotes

1 Health Extension Officers (HEOs) are universally comparable to physician assistant.

2 Community Health Workers (CHWs) are universally comparable to nurse aids.

## References

[1] Adachi K, Nielsen-Saines K, Klausner JD (2016). Chlamydia trachomatis infection in pregnancy: the global challenge of preventing adverse pregnancy and infant outcomes in Sub-Saharan Africa and Asia. Biomed Res Int..

[2] Saleska JL (2018). Use of antiretroviral therapy during pregnancy and adverse birth outcomes among women living with HIV-1 in low- and middle-income countries: a systematic review. J Acquir Immune Defic Syndr.

[3] Shava E (2019). High rates of adverse birth outcomes in HIV and syphilis co-infected women in Botswana. JAIDS J Acquir Immune Defic Syndr.

[4] Newman L (2013). Global Estimates of Syphilis in Pregnancy and Associated Adverse Outcomes: Analysis of Multinational Antenatal Surveillance Data. PLOS Medicine.

[5] Dingens AS (2016). Bacterial vaginosis and adverse outcomes among full-term infants: a cohort study. BMC pregnancy and childbirth.

[6] Olaleye AO (2020). Sexually transmitted infections in pregnancy – An update on chlamydia trachomatis and neisseria gonorrhoeae. Eur J Obstetr Gynecol Reprod Biol.

[7] Olson-Chen C, Balaram K, Hackney DN (2018). Chlamydia trachomatis and adverse pregnancy outcomes: meta-analysis of patients with and without infection. Matern Child Health J.

[8] Konadu DG (2019). Prevalence of vulvovaginal candidiasis, bacterial vaginosis and trichomoniasis in pregnant women attending antenatal clinic in the middle belt of Ghana. BMC Pregnancy and Childbirth.

[9] Gomez GB (2013). Untreated maternal syphilis and adverse outcomes of pregnancy: a systematic review and meta-analysis. Bulletin of the World Health Organization.

[10] Vallely LM (2021). Adverse pregnancy and neonatal outcomes associated with Neisseria gonorrhoeae: systematic review and meta-analysis. Sex Transm Infect.

[11] Vallely L.M (2018). Adverse pregnancy and neonatal outcomes associated with neisseria gonorrhoeae, mycoplasma genitalium, M. hominis, ureaplasma urealyticum and U. parvum: a systematic review and meta-analysis protocol. BMJ Open.

[12] WHO (2016). WHO recommendation on sexually transmitted infections testing in pregnancy.

[13] WHO regional office Western Pacific, second generation surveillance surveys of HIV, other STIs and risk behaviours in 6 [six] Pacific Island countries. 2006.

[14] Vallely A (2010). The prevalence of sexually transmitted infections in Papua New Guinea: a systematic review and meta-analysis. PloS one.

[15] Vallely A, et al. Point-of-care testing and treatment of sexually transmitted infections to improve birth outcomes in high-burden, low-income settings: study protocol for a cluster randomized crossover trial (the WANTAIM Trial, Papua New Guinea). Wellcome Open Res. 2019;4(53). 10.12688/wellcomeopenres.15173.2 PMC6979472 32030356

[16] PNG Obstetrics & gynaecology society, manual of standard managements in obstetrics and gynaecology for doctors, HEOs and nurses in Papua New Guinea. 2016.

[17] Severe L (2013). Rapid-testing technology and systems improvement for the elimination of congenital syphilis in Haiti: overcoming the "technology to systems gap". J Sex Transm Dis.

[18] Acharya K (2020). Availability and readiness to provide sexually transmitted infections and HIV testing and counselling services in Nepal: evidence from comprehensive health facility survey. BMJ Open.

[19] Lama TP (2020). Assessment of facility and health worker readiness to provide quality antenatal, intrapartum and postpartum care in rural Southern Nepal. BMC Health Serv Res.

[20] Yadav H (2021). Availability of essential diagnostics in ten low-income and middle-income countries: results from national health facility surveys. The Lancet Global Health.

[21] Kruk ME (2018). High-quality health systems in the Sustainable Development Goals era: time for a revolution. The Lancet Global Health.

[22] Sheffel A, Karp C, Creanga AA (2018). Use of service provision assessments and service availability and readiness assessments for monitoring quality of maternal and newborn health services in low-income and middle-income countries. BMJ Global Health.

[23] Tunçalp Ӧ (2015). Quality of care for pregnant women and newborns-the WHO vision. BJOG : Int J Obstetr Gynaecol.

[24] Razzaq A, Raynes-Greenow C, Alam A (2021). Barriers to uptaking HIV testing among pregnant women attending antenatal clinics in low- and middle-income countries: a systematic review of qualitative findings. Aus N Z J Obstetr Gynaecol.

[25] PNG national department of health, PNG health plan 2011-2020. 2010.

[26] PNG national AIDS council secretariat, Papua New Guinea national STI and HIV strategy 2018–2022. 2017, GoPNG: Port Moresby.

[27] PNG national department of health, health information systems. 2019.

[28] Badman SG (2016). A novel point-of-care testing strategy for sexually transmitted infections among pregnant women in high-burden settings: results of a feasibility study in Papua New Guinea. BMC Infect Dis.

[29] PNG national department of health, 2018 STI/HIV and AIDS program annual report. 2019: Port Moresby.

[30] WHO, service availability and readiness assessment (SARA): reference manual. 2015. 2.2.

[31] The World Bank Group. Papua New Guinea country indicators. 2020 January 29 2021]; Available from: https://data.worldbank.org/country/papua-new-guinea.

[32] Grundy, J., Dakulala, P, Wai, K, Maalsen, A and Whittaker, M,, Independent State of Papua New Guinea health system review. 2019, world health organization. Regional office for South-East Asia,.

[33] PNG national department of health, national health pan 2021-2030. 2021.

[34] PNG national department of health, sector performance annual review- province and district health profiles (2018). 2018.

[35] PNG national department of health, Sector Performance Annual Review (SPAR) report 2019. 2020.

[36] Hou, X.K., M. Mahmud; Pulford, Justin; Saweri, Olga; Demir, Ibrahim; Haider, Rifat; Ahmed, Shakil;, Service delivery by health facilities in Papua New Guinea : report based on a countrywide health facility survey (English). . 2018, The WorldBank Group.

[37] O'Neill K (2013). Monitoring service delivery for universal health coverage: the Service Availability and Readiness Assessment. Bull World Health Organ.

[38] Spiegel DA (2017). Retrospective review of Surgical Availability and Readiness in 8 African countries. BMJ Open.

[39] WHO, Health workforce requirements for universal health coverage and the sustainable development goals, in human resources for health observer. 2016.

[40] Leslie HH (2017). Service readiness of health facilities in Bangladesh, Haiti, Kenya, Malawi, Namibia, Nepal, Rwanda, Senegal, Uganda and the United Republic of Tanzania. Bulletin of the World Health Organization.

[41] WHO, WHO recommendation on antenatal care for a positive pregnancy experience. 2016: Luxembourg. 28079998

[42] WHO, Pregnancy, childbirth, postpartum and newborn care: a guide for essential practice. 2015, WHO. p. 184. 26561684

[43] Kaiser HF (1974). An index of factorial simplicity. Psychometrika.

[44] Tabachnick, B.F., L,, Using multivariate statistics,. 6th Ed. ed. 2007, : Boston, MA, USA: Pearson education.

[45] Jackson EF (2015). Estimation of indices of health service readiness with a principal component analysis of the Tanzania service provision assessment survey. BMC Health Serv Res.

[46] Henson RK, Roberts JK (2006). Use of exploratory factor analysis in published research: common errors and some comment on improved practice. Educ Psychol Meas.

[47] Bintabara D, Ernest A, Mpondo B (2019). Health facility service availability and readiness to provide basic emergency obstetric and newborn care in a low-resource setting: evidence from a Tanzania National Survey. BMJ open.

[48] Gage AJ, Ilombu O, Akinyemi AI (2016). Service readiness, health facility management practices, and delivery care utilization in five states of Nigeria: a cross-sectional analysis. BMC Pregnancy and Childbirth.

[49] Andrew EVW (2014). Factors Affecting Attendance at and Timing of Formal Antenatal Care: Results from a Qualitative Study in Madang, Papua New Guinea. PLOS ONE.

[50] Wilunda C (2017). Barriers to utilisation of antenatal care services in South Sudan: a qualitative study in Rumbek North County. Reprod Health.

[51] Mueller I (1998). The effect of distance from home on attendance at a small rural health centre in Papua New Guinea. Int J Epidemiol.

[52] Thomason JA, Kolehmainen-Aitken R-L (1991). Distribution and performance of rural health workers in Papua New Guinea. Soc Sci Med.

[53] Matsumoto M, Inoue K, Kajii E (2010). Policy implications of a financial incentive programme to retain a physician workforce in underserved Japanese rural areas. Soc Sci Med.

[54] Bärnighausen T, Bloom DE (2009). Financial incentives for return of service in underserved areas: a systematic review. BMC Health Serv Res.

[55] WHO, Improving retention of health workers in rural and remote areas: case studies from the WHO South-East Asia Region. 2020, WHO: New Delhi.

[56] Szabo S (2020). Health workforce demography: a framework to improve understanding of the health workforce and support achievement of the Sustainable Development Goals. Hum Resour Health.

[57] WHO, World Health Statistics 2015. 2015: Geneva.

[58] Finlayson K, Downe S (2013). Why do women not use antenatal services in low- and middle-income countries? A meta-synthesis of qualitative studies. PLoS Med.

[59] Miteniece E (2017). Barriers to accessing adequate maternal care in Central and Eastern European countries: A systematic literature review. Soc Sci Med.

[60] Mannava P (2015). Attitudes and behaviours of maternal health care providers in interactions with clients: A systematic review. Global Health.

[61] Kyei-Nimakoh M, Carolan-Olah M, McCann TV (2017). Access barriers to obstetric care at health facilities in sub-Saharan Africa—a systematic review. Syst Rev.

[62] Arsenault C (2018). Equity in antenatal care quality: an analysis of 91 national household surveys. The Lancet Global Health.

[63] Kruk ME (2016). Quality of basic maternal care functions in health facilities of five African countries: an analysis of national health system surveys. The Lancet Global Health.

[64] Gauthier B, Wane W (2011). Bypassing health providers: the quest for better price and quality of health care in Chad. Soc Sci Med.

[65] Rao KD, Sheffel A (2018). Quality of clinical care and bypassing of primary health centers in India. Soc Sci Med.

[66] Macarayan EK (2018). Assessment of quality of primary care with facility surveys: a descriptive analysis in ten low-income and middle-income countries. The Lancet. Global health.

[67] McCarthy EA (2017). Quality improvement intervention to increase adherence to ART prescription policy at HIV treatment clinics in Lusaka, Zambia: A cluster randomized trial. PLoS One.

[68] Larsen GL (2004). Antenatal care in Goroka: issues and perceptions. P N G Med J.

[69] Myer L, Harrison A (2003). Why do women seek antenatal care late? Perspectives from rural South Africa. J Midwifery Women's Health.

[70] Mason L (2015). Barriers and facilitators to antenatal and delivery care in western Kenya: a qualitative study. BMC Pregnancy and Childbirth.

[71] Kananura RM (2017). Persisting demand and supply gap for maternal and newborn care in eastern Uganda: a mixed-method cross-sectional study. Reprod Health.

[72] Mitton C, Donaldson C (2004). Health care priority setting: principles, practice and challenges. Cost Eff Resour Alloc.

[73] Adawiyah R.a (2022). Supply-side readiness to deliver HIV testing and treatment services in Indonesia: Going the last mile to eliminate mother-to-child transmission of HIV. PLOS Global Public Health.

[74] Bintabara D (2021). Does facility readiness promote high-quality of provider-initiated HIV testing and counseling to pregnant women? A national survey for improving policy of prevention of mother-to-child transmission of HIV in Tanzania. AIDS Res Ther.

[75] Machekera S (2021). Strategic options for syphilis control in Papua New Guinea- impact and cost-effectiveness projections using the syphilis interventions towards elimination (SITE) model. Infect Dis Model.

